# Evidence of spontaneous selfing and disomic inheritance in *Geranium robertianum*

**DOI:** 10.1002/ece3.7677

**Published:** 2021-06-03

**Authors:** Fabienne Van Rossum, Olivier Raspé, Filip Vandelook

**Affiliations:** ^1^ Meise Botanic Garden Meise Belgium; ^2^ Service général de l'Enseignement supérieur et de la Recherche scientifique Fédération Wallonie‐Bruxelles Brussels Belgium; ^3^ Biology Department Philipps Universität Marburg Marburg Germany; ^4^ Present address: School of Science Mae Fah Luang University Chiang Rai Thailand

**Keywords:** autonomous selfing, disomic inheritance, duplicate loci, *Geranium robertianum*, microsatellites

## Abstract

Knowing species’ breeding system and mating processes occurring in populations is important not only for understanding population dynamics, gene flow processes, and species' response to climate change, but also for designing control plans of invasive species. *Geranium robertianum*, a widespread biennial herbaceous species showing high morphological variation and wide ecological amplitude, can become invasive outside its distribution range. A mixed‐mating system may be expected given the species’ floral traits. However, autonomous selfing is considered as a common feature. Genetic variation and structure, and so population mating processes, have not been investigated in wild populations. We developed 15 polymorphic microsatellite markers to quantify genetic variation and structure in *G*. *robertianum*. To investigate whether selfing might be the main mating process in natural conditions, we sampled three generations of plants (adult, F1, and F2) for populations from the UK, Spain, Belgium, Germany, and Sweden, and compared open‐pollinated with outcrossed hand‐pollinated F2 progeny. The highly positive Wright's inbreeding coefficient (*F*_IS_) values in adults, F1, and open‐pollinated F2 progeny and the low *F*_IS_ values in outcross F2 progeny supported autonomous selfing as the main mating process for *G. robertianum* in wild conditions, despite the presence of attractive signals for insect pollination. Genetic differentiation among samples was found, showing some western–eastern longitudinal trend. Long‐distance seed dispersal might have contributed to the low geographic structure. Local genetic differentiation may have resulted not only from genetic drift effects favored by spontaneous selfing, but also from ecological adaptation. The presence of duplicate loci with disomic inheritance is consistent with the hypothesis of allotetraploid origin of *G. robertianum*. The fact that most microsatellite markers behave as diploid loci with no evidence of duplication supports the hypothesis of ancient polyploidization. The differences in locus duplication and the relatively high genetic diversity across *G. robertianum* range despite spontaneous autonomous selfing suggest multiple events of polyploidization.

## Graphical abstract

Genetic analyses based on 15 polymorphic microsatellite markers supported autonomous selfing as the main mating process for *Geranium robertianum* in wild populations, despite the presence of attractive signals for insect pollination. Genetic differentiation showed some longitudinal trend. Long‐distance seed dispersal might have contributed to the low geographic structure. Local genetic differentiation may have resulted not only from genetic drift effects favored by spontaneous selfing, but also from ecological adaptation. The presence of duplicate loci with disomic inheritance is consistent with the hypothesis of allotetraploid origin of *G. robertianum*.

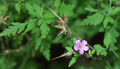

## 1 INTRODUCTION

The breeding system in Angiosperms can vary from autogamy (self‐fertilization) to strict allogamy (obligate outcrossing). Strict allogamy may also evolve into a heteromorphic self‐incompatibility system preventing selfing or into dioecy (Charlesworth, 2006; Richards, 1997). Autogamy can allow for purging deleterious recessive alleles by natural selection (Charlesworth & Charlesworth, 1987; Goodwillie et al., 2005) and facilitate colonization of new territories for pioneer species or occurrence in extreme or unpredictable habitats where pollinators are scarce or absent (Barrett, 2003; Hartfield et al., 2017; Kalisz & Vogler, 2003). However, it can reduce effective genome recombination and within‐population genetic diversity (e.g., Bomblies et al., 2010; Jullien et al., 2019; Nordborg, 2000). Obligate outcrossing represents an advantage by mixing gene pools, increasing genetic diversity, and preventing inbreeding depression (Arista et al., 2017; Charlesworth, 2006), but it can require pollinating vectors, such as insects, birds, or bats, and a sufficient number of compatible mates or extensive gene flow between populations for ensuring reproductive success (Berjano et al., 2013; Menz et al., 2011). Retaining facultative self‐pollination, in particular delayed autonomous selfing, can offer reproductive assurance when outcrossing has not occurred in case of limited pollinator service (Busch & Delph, 2012; Kalisz & Vogler, 2003). Pollinator service may be limited in fragmented habitats or in case of temporary unfavorable environmental conditions (Arista et al., 2017; Goodwillie & Weber, 2018). Therefore, a lot of species are characterized by a mixed‐mating system to guarantee seed production despite a risk of inbreeding depression in the progeny (Goodwillie et al., 2005, 2010; Kalisz et al., 2004).

Outcrossing species usually possess attractive floral traits for pollinators, for example, a high number of colored flowers and nectar reward, whereas autonomous selfers often have reduced floral display and nectar reward (Bartoš et al., 2020; Goodwillie et al., 2010; Sicard & Lenhard, 2011). Knowing species’ breeding system and quantifying mating processes (outcrossing and selfing rates), which occur in populations, are important for understanding population dynamics, gene flow processes, and potential species' response to climate change (Charlesworth, 2006; Razanajatovo et al., 2020). They are also important for designing conservation recovery plans of endangered species and control plans of invasive exotic species (Barrett, 2010; Dudash & Murren, 2008). For instance, small populations of species with a self‐incompatibility system require a high number of compatible mates for successful demographic and genetic restoration, whereas inbreeding issues may be found for species with a mixed‐mating system, requiring genetic rescue of small populations (e.g., Menges, 2008; Olivieri et al., 2016; Van Rossum, Destombes et al., 2021). Autonomous selfers may easily produce seeds and naturalize, and may therefore become potentially invasive outside their distribution range (Antoń & Denisow, 2018; Razanajatovo et al., 2016). Exclusion and pollination experiments can give insights on whether species are self‐compatible or self‐incompatible (e.g., Bartoš et al., 2020), but genetic studies using molecular markers can allow for quantifying outcrossing rates, inbreeding levels, pollen dispersal processes, and genetic diversity and structure in wild populations (e.g., Arista et al., 2017; Bomblies et al., 2010; Charlesworth, 2006; Gelmi‐Candusso et al., 2017; Jacquemart et al., 2021).

*Geranium robertianum* L. (Geraniaceae) is a common, biennial(–annual), ruderal herb and is highly variable morphologically. The species shows a wide ecological amplitude, mainly occurring in woodlands and hedge banks, but also in various open habitats, such as grasslands, wastelands, railway banks, skeletal soils, and walls, on calcareous and acidic soils (Tofts, 2004; Vandelook & Van Assche, 2010; Wierzbicka et al., 2014). It is widely spread in its native distribution area in Europe, and naturalized in temperate regions of many other continents, where it can become invasive (Tofts, 2004). Individual plants bear between 10 and 300 pink flowers (12–17 mm diameter), usually slightly protandrous, sometimes homogamous or protogynous (Bertin, 2001; Tofts, 2004). The dehiscing of the five inner anthers usually precedes the lengthening of the style and stigma receptivity. When the inner stamens wither, the fiver outer anthers move to the center of the flower around the style and dehisce (Knuth, 1908; Tofts, 2004). Flowers stay open for two to five days (Tofts, 2004; F. Vandelook, *personal observation*), which is similar to other *Geranium* species (e.g., Willson et al., 1979). Generally, five seeds per fruit are produced (Tofts, 2004). Flowers produce nectar and are visited by insects, in particular butterflies, Syrphid flies, wild bees, and honey bees (Endress, 2010; Tofts, 2004; Yeo, 1973), suggesting outcrossing. Self‐fertilization is, however, possible, as stigmas during elongation can be covered with pollen of the inner whorl of stamens before possible outcrossing events, and when the stigmas standing above the dehiscing outer anthers recurve (Knuth, 1908; Tofts, 2004), allowing for prior and delayed autonomous selfing. Autonomous selfing has been considered as a common feature (Bertin, 2001; Yeo, 1973, 1985). Consequently, mixed mating likely occurs in *G. robertianum*. However, population mating processes have never been investigated in the field using codominant molecular markers to estimate genetic variation and inbreeding levels. Besides, plants only reproduce by seeds, which are dispersed not only at short distances by carpel projection but also at long distances by epizoochory (Tofts, 2004; Yeo, 1973). As a result, genetic variation and structure patterns may be contrasted according to mating processes and short‐ and long‐distance seed dispersal (e.g., Bomblies et al., 2010; Gelmi‐Candusso et al., 2017; Helsen et al., 2015; Jacquemart et al., 2021). Moreover, due to its wide distribution range combined with a wide ecological amplitude, *G. robertianum* appears as an interesting model for studying local adaptation and response to climate change (Hoffmann & Sgrò, 2011; Wierzbicka et al., 2014). Therefore, we developed polymorphic microsatellite markers to quantify genetic variation and structure in *G. robertianum*. To investigate whether selfing might be the main mating process in natural conditions, we sampled three generations of plants (adult, F1, and F2) for populations from the UK, Spain, Belgium, Germany, and Sweden, and progeny obtained from outcrossed hand‐pollinated were compared with progeny in open‐pollinated conditions.

## 2 MATERIALS AND METHODS

### 2.1 Study populations and sampling

**FIGURE 1:**
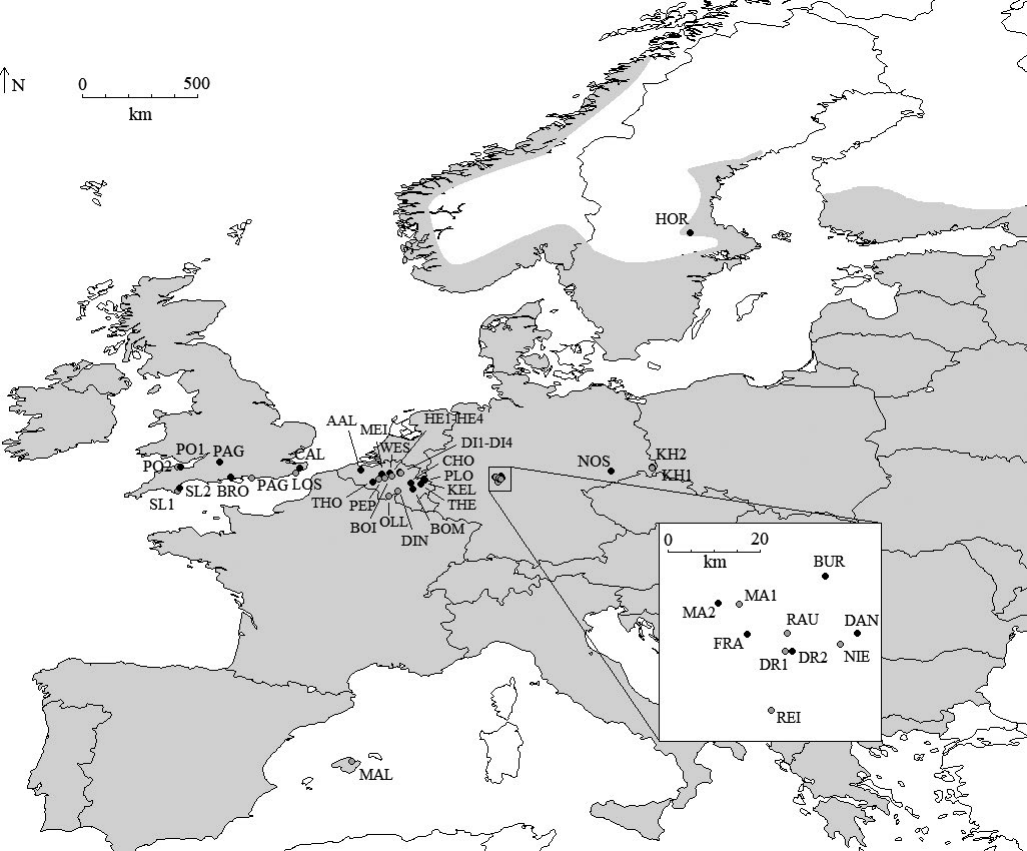
Location of the 43 populations of *Geranium robertianum* (gray dot: open habitat; black dot: forest habitat) sampled in the UK, Spain, Belgium, Germany, and Sweden. For population codes, see Table 1. The approximate native distribution range is indicated in light gray (modified from Hultén & Fries, 1986)

To cover a wide ecological amplitude and geographic range of *G. robertianum*, 43 populations were selected from various calcareous or acidic habitats (e.g., forests, forest edges, grasslands, railway banks, sandy and shingle beaches), from the UK, Spain, Belgium, Germany, and Sweden (Figure 1, Table 1). Populations varied in size, ranging from about 20 flowering individuals up to hundreds of individuals (F. Vandelook, *unpublished data*). Leaves were collected from a total of 191 individuals (adults, and F1 and F2 seed progeny) and dried in silica gel. Sampling occurred during the peak of flowering and pollinator activity, between mid‐June and mid‐August. F1 progeny was obtained from germination of seeds collected in wild populations on different maternal plants separated at least 1 meter from each other. F1 plants from eight populations from UK and Germany were grown (1) in the Botanical Garden of the Phillips‐Universität Marburg (Germany) in 2013 until flowering and fruiting, with plants randomly distributed (but covered with shade nets, which might have reduced insect pollination); F2 seed progeny was obtained from germination of seeds collected on the F1 plants after open pollination (F2o); (2) in a nonheated greenhouse at Meise Botanic Garden (Belgium) in late May‐June 2019, where F2 seed progeny was obtained after outcrosses (F2c) between F1 plants (Table 2) or selfing (F2s, but only for two maternal plants and four progenies in total). For the crossing experiment, flowers were emasculated before anthesis and bagged. Stigmas were hand‐pollinated with pollen when they were receptive on two consecutive days and rebagged until ripe seeds could be collected, after three to four weeks.

**TABLE 1:** Details for 43 populations of *Geranium robertianum*: location, country (UK: United Kingdom, SP: Spain, BE: Belgium, GE: Germany, SW: Sweden) geographic coordinates, habitat type, soil pH (measured in 1:1 soil‐distilled water mixture), and *n*: sample size (A: adults; F1: F1 seed progeny; F2o, F2s, and F2c: F2 seed progeny, obtained from open, self, and outcross pollination, respectively; in bold: samples used for microsatellite development)

Population	Locality	Country	Latitude (N)	Longitude (W or E)		Habitat type	Soil pH	*n*				
								A	F1	F2o	F2c	F2s
PAG	Pagham	UK	50°45'50''	0°44'35''	W	Shingle beach	8.2		2			
LOS	Littlestone‐on‐Sea, Kent	UK	50°58'18''	0°57'52''	E	Sandy beach	7.0		4			
ELH	Elham	UK	51°09'45''	1°06'24''	E	Hedge bank	6.8		2			
BRO	Brockenhurst	UK	50°49'39''	1°34'17''	W	Hedge and forest	7.0		2			
CAL	Calne	UK	51°25'07''	1°59'36''	W	Forest			3	4	5	
PO1	Porlock	UK	51°13'05''	3°37'31''	W	Shingle beach	8.3		2			
PO2	Porlock	UK	51°13'06''	3°37'46''	W	Hedge and forest near the beach	6.5		3			
SL1	Slapton	UK	50°17'17''	3°38'41''	W	Shingle	6.2		2			
SL2	Slapton	UK	50°17'29''	3°39'10''	W	Hedge bank	7.1		3	5	8	
MAL	Mallorca	SP	39°45'37''	3°09'20''	E	Park near forest			2			
AAL	Aalter	BE	51°05'04''	3°29'34''	E	Forest		**1**				
THO	Thoricourt	BE	50°37'00"	3°57'11"	E	Forest fringe		**1**				
PEP	Pepingen	BE	50°44'41"	4°11'59"	E	Ruderal grassland		**1**				
MEI	Meise	BE	50°55'42''	4°19'31''	E	Forest		**4**				
BOI	Boistfort	BE	50°47'35"	4°24'57"	E	Ruderal grassland on roadside		**1**				
WES	Wespelaar	BE	50°57'41''	4°38'52''	E	Forest fringe		**1**				
HE1	Heverlee, Leuven	BE	50°51'56''	4°41'22''	E	Forest			2			
HE2	Heverlee, Leuven	BE	50°51'48''	4°42'20''	E	Railway bank	6.9		3			
DI1	Molenstede	BE	51°00'38''	5°01'53''	E	Forest		**1**				
DI2	Molenstede, Diest	BE	51°01'00''	5°01'59''	E	Forest		21	3			
DI3	Diest	BE	50°58'23''	5°02'02''	E	Ruderal grassland on roadside		**1**				
DI4	Diest	BE	50°59'36''	5°02'53''	E	Railway bank	8.1		3			
OLL	Olloy‐sur‐Viroin	BE	50°04'08"	4°36"22"	E	Ruderal grassland on schists	4.1	**1**				
DIN	Dinant	BE	50°16'29"	4°56"03"	E	Ruderal roadside on limestone	7.5	**1**				
CHO	Chokier	BE	50°35'34"	5°26'30"	E	Forest on limestone	7.6	**1**				
BOM	Bomal‐sur‐Ourthe	BE	50°21'40"	5°31'02"	E	Forest fringe on limestone	7.6	**1**				
THE	Theux	BE	50°32'28"	5°49'51"	E	Forest on acidic soil		**1**				
PLO	Plombières	BE	50°44'10"	5°58'03"	E	Forest		**1**				
KEL	Kelmis	BE	50°41'43"	5°59'17"	E	Forest fringe		**1**				
MA2	Marbach, Marburg	GE	50°49'29''	8°43'56''	E	Forest	6.1		3			
MA1	Marburg	GE	50°49'21''	8°46'22''	E	Railway bank	7.7		3			
FRA	Frauenberg, Marburg	GE	50°45'49''	8°47'19''	E	Forest	5.8		1			
REI	Reiskirchen, Giessen	GE	50°36'44''	8°50'11''	E	Roadside on schists	8.3		3	4	10	
DR1	Dreihausen, Ebsdorfergrund	GE	50°43'47''	8°51'58''	E	Quarry	7.5		1			
RAU	Rauischholzhausen, Ebsdorfergrund	GE	50°45'48''	8°52'07''	E	Roadside	7.0		3	3	5	2
DR2	Dreihausen, Ebsdorfergrund	GE	50°43'48''	8°52'36''	E	Forest	4.3		2			
BUR	Burgholz, Kirchhain	GE	50°52'40''	8°56'44''	E	Forest	4.8		2			
NIE	Nieder‐Ofleiden, Homberg	GE	50°44'36''	8°58'27''	E	Railway bank	8.3		3	4	3	
DAN	Dannenrod, Homberg	GE	50°45'48''	9°00'25''	E	Forest	5.4		3	7	9	
NOS	Nossen	GE	51°03'27''	13°13'36''	E	Forest	6.7		1			
KH1	Königshain	GE	51°11'17''	14°50'54''	E	Railway bank	6.8		3	6	1	
KH2	Königshain	GE	51°11'28''	14°50'58''	E	Forest fringe	6.7		3	2	2	2
HOR	Horndal	SW	60°19'14''	16°19'14''	E	Forest			3			

**TABLE 2:** Summary of crosses between populations and number of genotyped seed progeny per cross (for population codes, see Table 1)

Maternal plant	Pollen donor							
	CAL	DAN	KH1	KH2	NIE	RAU	REI	SL2
CAL			1	1		3		
DAN					6	2		1
KH1						1		
KH2		2						
NIE			1					2
RAU				2	3			
REI	2		5					3
SL2		3	2				3	

### 2.2 DNA analyses

#### 2.2.1 DNA extraction

DNA was isolated from ca. 15–25 mg of dried leaf material for 191 samples using a CTAB method (Doyle & Doyle, 1990). We estimated the concentration of genomic DNA extracts using the Qubit Quantitation Platform (Invitrogen), which was standardized to 2 ng/μl.

#### 2.2.2 Nuclear microsatellite primer development and multiplexing

Nuclear microsatellites were developed by Genoscreen (Lille, France) as described in Van Rossum, Destombes et al. (2021). Genomic DNA of 15 individuals was used (Table 1). A microsatellite library was developed using 1 μg from an equimolar DNA pool of 10 individuals through 454 GS‐FLX Titanium pyrosequencing of a DNA library enriched for AG, AC, AAC, AAG, AGG, ACG, ACAT, and ATCT repeat motifs (Malausa et al., 2011). PCR products were purified and quantified, and GsFLX library was then constructed and sequenced on a GS‐FLX PTP. The selection of sequences with target microsatellites was performed using QDD with the parameters set by default (Meglécz et al., 2010). The sequence reads were submitted to the NCBI Sequence Read Archive (SRA) database under the accession number PRJNA694498. Among 38,206 raw sequence reads, 4,030 sequences comprised a microsatellite motif, from which 461 primer pairs were designed on flanking regions.

For biological validation, a total of 47 primer pairs showing a high number of repeats (at least 9) and covering a wide range of PCR product sizes (from 101 to 316 bp) were tested for amplification on eight DNA samples. PCR amplifications were carried out in 10 μl reactions containing 20 ng of template DNA, 1× reaction buffer, 37.5 pmol MgCl2, 6 pmol dNTP, 10 pmol of each primer, and 0.5 U Taq polymerase (FastStart—Roche Diagnostics). The PCR cycling consisted of an initial denaturation at 95°C for 10 min, followed by 40 cycles: denaturation at 95°C for 30 s, annealing at 55°C for 30 s and extension at 72°C for 1 min, and a final extension at 72°C for 10 min. Primer pairs were discarded after migration of PCR products on 2% agarose gel electrophoresis when they did not amplify or gave multiple fragments. As a result, 39 primer pairs were validated from which 24 microsatellite loci that showed good amplification for all individuals and still covered a wide range of PCR product sizes (from 101 to 316 bp) were selected for polymorphism study on 15 DNA samples. PCR amplifications were performed with the same conditions than previously but with labeled primers (Di‐repeat +tail Applied Biosystems). Each PCR product (diluted at 1:50 with dH2O) was mixed with Hi‐Di^TM^ Formamide (Life Technologies, Carlsbad, California, USA) and GeneScan^TM^ 500 LIZ® Dye Size Standard (Applied Biosystems). Fragments were migrated on a 3730XL DNA capillary sequencer (Applied Biosystems). Alleles were scored using the microsatellite plugin in Geneious 11.1.2 (Biomatters). Finally, 15 polymorphic and interpretable markers (Table 3) were selected and three multiplexes were developed using Multiplex Manager v1.2 (Holleley & Geerts, 2009) and subsequently optimized. All individuals were genotyped using the same protocol.

**TABLE 3:** Characteristics of 15 microsatellite markers developed in *Geranium robertianum*. For each marker (and duplicate loci in GER17, GER35, GER42, GER45, and GER47 indicated as A and B), the forward and reverse sequences, repeat type, size of the original fragment (bp), number of alleles (*An*), allele size range, multiplex number, ﬂuorescent dye, primer amount used in the multiplex PCR (pmol), and null allele frequency (with their 95% highest posterior density intervals) are given

Locus name	Primer sequence (5’–3’)	Repeat motif	*An*	Size range (bp)	Multiplex number	Dye	Primer amount (pmol)	Null allele frequency
GER07	F: AGTGGCTTTTACCGAACACG	(tct)13	16	96–153	1	6‐FAM	1.6	0.010
	R: TGAAGGTGTTTGAGGCAACA							(0.000–0.024)
GER29	F: CCTTTGTGTTTGATAGCATTTAAGA	(ctt)10	7	93–117	1	VIC	4	0.019
	R: AAATTGAGCGTTGTCGCATA							(0.000–0.039)
GER08	F: ATATAAACCCCAAGACCGCC	(ctt)12	13	260–296	1	NED	1	0.026
	R: TCCTCCGAATGAGACCTCTG							(0.000–0.048)
GER45	F: CGAAAACCCTAGAACCGACA	(aga)9	6	113–128	1	NED	1	
	R: CATGGTCGTGGTTCAGTTTG		3	A: 113–119				0.010
								(0.000–0.025)
			5	B: 116–128				0.028
								(0.003–0.056)
GER17	F: GGGTCATTTTCGACCTTTCA	(ag)11	10	142–166	1	PET	1.6	
	R: AGACGATGGGTCGATTGAAG		4	A: 142–148				0.015
								(0.000–0.033)
			7	B: 148–166				0.031
								(0.006–0.060)
GER26	F: CTTTCCTCTTGTGCTTCGCT	(ag)10	4	151–157	2	6‐FAM	4	0.133
	R: GATTCAAACAAGCCTCTGCC							(0.087–0.184)
GER30	F: AGAATATGACCAATCCAACACC	(ctt)10	7	96–120	2	6‐FAM	4	0.017
	R: CTCTTGGTAGCCAATGGAGG							(0.001–0.037)
GER05	F: ATCTTAGCGCTTCCTCTCCC	(ct)13	11	168–194	2	VIC	1.8	0.013
	R: TCCGAAGCTGGAGCTCTATT							(0.000–0.029)
GER42	F: AATGCTGAAGCTGTCCCCTA	(tc)9	5	119–137	2	NED	1.6	
	R: CCCAAGAACAGTAGTAAGAGAATTTG		3	A: 119–133				0.016
								(0.000–0.037)
			2	B: 135–137				0.017
								(0.000–0.041)
GER23	F: AAAGTCACAACTCGGTCAATAGC	(tct)10	5	199–220	2	PET	2.24	0.004
	R: GTGGGATTCTGGAAGCTGAA							(0.000–0.017)
GER41	F: TCGTCTTGAGGAAGAAGCGT	(ttc)9	13	138–174	2	PET	1.26	0.007
	R: CATGCTCGCAGAGTAGCCTT							(0.000–0.022)
GER27	F: TGCAAAGTCTGTCAACGTCA	(ct)10	5	139–147	3	6‐FAM	1.6	0.003
	R: GTCTCACAGACTTCCCTCGC							(0.000–0.013)
GER47	F: CAAGGAAACTCGGGATCATCT	(tc)9	7	107–125	3	VIC	1.8	
	R: AGAACGAGGCGGGATCTAAT		4	A: 107–113				0.021
								(0.000–0.044)
			5	B: 111–125				0.027
								(0.003–0.055)
GER38	F: TGGTTGTCTCTGAAGCACTCA	(ctt)9	8	136–157	3	NED	6	0.020
	R: CCCAATATTTACCATTTTGTCTTG							(0.000–0.040)
GER35	F: AAGCGATACACGAATGGAAAA	(ga)9	10	188–208	3	PET	5	
	R: AGAAAATACGCACCGTGGAG		3	A: 188–192				0.019
								(0.000–0.042)
			8	B: 192–208				0.012
								(0.000–0.029)

### 2.3 Data analysis

#### 2.3.1 Null alleles and independence of the loci

Each locus was checked for potential null alleles using INEST 2.2 (Chybicki & Burczyk, 2009). We conducted the Bayesian approach (IIM) with 10^6^ Markov Chain Monte Carlo iterations, of which the first 10^5^ were discarded as burn‐in phase to test two models: a full model (*nfb*, including null alleles, inbreeding, and genotyping failures) and a model (*nb*) where there was no inbreeding. The best fitting model corresponded to the lowest value of the deviation information criterion (DIC). INEST also estimated null allele frequencies for each locus with their 95% highest posterior density intervals (HPDI). To assess the independence of the loci, a test for genotypic disequilibrium was performed between pairs of loci with sequential Bonferroni‐type correction (Rice, 1989) on adults and on F1 progeny using FSTAT version 2.9.4 (Goudet, 2003).

#### 2.3.2 Genetic variation within generations

Expected heterozygosity (*H*_e_) and Wright's inbreeding coefficient (*F*_IS_), corrected for small sample size, were calculated for each locus and for each generation (adults, F1 progeny, and F2 progeny) using FSTAT. We tested the significance of the *F*_IS_ values (for each locus and over all loci) by randomization tests and sequential Bonferroni‐type correction. Differences in *H*_e_ and *F*_IS_ between F1, open‐pollinated, and outcross F2 progeny were tested for the eight populations used for the crossing experiment (Table 2) by the Wilcoxon matched‐pairs tests by locus using STATISTICA version 12.

#### 2.3.3 Population genetic structure at a wide geographic scale

To investigate population genetic structure patterns, we performed a principal coordinate analysis (PCoA) based on a standardized distance matrix using GenAlEx 6.5 (Peakall & Smouse, 2012) and Bayesian clustering analyses using STRUCTURE version 2.3.4 (Pritchard et al., 2000) on adults, F1 progeny, and open‐pollinated F2 progeny. For STRUCTURE analyses, we computed 15 runs for *K* = 1 to 10 clusters, using an admixture ancestry model with correlated allele frequencies and no prior population information, run length of burn‐in period of 10^6^ iterations, and 2 10^6^ Markov Chain Monte Carlo replications. The most likely number of *K* clusters was inferred as described in Evanno et al. (2005) after running STRUCTURE HARVESTER (Earl & vonHoldt, 2012). The most likely estimated membership (*Q*) values of the 15 independent runs computed with CLUMPP version 1.1.2 (Jakobsson & Rosenberg, 2007) were visualized as a bar plot.

## 3 RESULTS

### 3.1 Loci and scored alleles

Out of the 15 primer pairs, 10 could be interpreted to amplify diploid loci. Five primer pairs (GER17, GER35, GER42, GER45, and GER47) showed two to four peaks ascribed to different alleles (Figure S1). From the genotyping of the F2 progeny obtained by outcrosses (F2c) and of their maternal and paternal plants, the amplified regions for each primer pair could be interpreted as corresponding to two duplicate loci (Table 3), not overlapping for GER42, but overlapping for the four other markers (Figure S1). However, the higher size of the peak allowed us to identify when two overlapping alleles occurred in both loci. For GER35, only one (rare) allele was found in both loci, and separating the two loci was easy. For GER17, GER45, and GER47, it can be difficult to distinguish both loci in some genotypes without data on maternal and paternal plants together with their progeny, and so we recommend not using them unless performing paternity analyses or cross experiments. For MAL population (Mallorca, Spain), GER17 and GER47 did not appear to be duplicated and some other markers did not amplify.

We scored two to 16 alleles in the 20 loci for a total of 133 alleles (Table 3). Five loci showed evidence for null alleles as 95% HPDI differed from 0, but only GER26 showed a high null allele frequency (0.133; 95% HPDI: 0.087–0.184; Table 3). There was significant genotypic disequilibrium between 15 and 6 of the 190 pairs of loci after sequential Bonferroni correction (*p* < .05) for adults and F1 progeny, respectively.

### 3.2 Genetic variation within generations

Expected heterozygosity (*H*_e_) values per locus ranged from 0.037 to 0.901 with a mean of 0.550 for adults and F1 progeny collected in wild populations (Table 4), and were similar between F1, open pollination (F2o), and outcross (F2c) F2 progeny (Wilcoxon test *Z* = 0.50–1.72, *p* ≤ .085). Wright's inbreeding coefficient (*F*_IS_) values significantly (*p* < .05) differed from the Hardy–Weinberg expectations, with a deficit of heterozygotes for almost all loci, except for (F2c) progeny, for which *F*_IS_ values were not significant or significantly negative (Table 4), and significantly lower than F1 and F2o progeny (*Z* = 3.72, *p* < .001). *F*_IS_ values were slightly but significantly lower (*Z* = 2.33, *p* = .020) for the F2o progeny obtained from open pollination of randomly distributed plants from eight populations, compared with F1 progeny collected in the same eight wild populations (mean *F*_IS_ = 0.668 and 0.768, respectively), suggesting the occurrence of some, however, limited, outcrossing. The IIM analysis indicated that inbreeding contributed to the positive *F*_IS_ values (lowest DIC for the *nfb* model). Population DI2, for which 21 adults were sampled across the whole population, showed 11 different multilocus genotypes, with 1–9 individuals sharing the same multilocus genotype, *H*_e_ = 0.318 and *F*_IS_ = 0.656 (significantly positive, *p* < .05).

**TABLE 4:** Within‐population genetic estimates per locus and per generation (wild: samples from wild populations, grouping adults and F1 progeny; A: adults; F1: F1 seed progeny; F2o, F2s, and F2c: F2 seed progeny, obtained from open, self, and outcross pollination, respectively) for *Geranium robertianum* at 20 nuclear microsatellite loci: expected heterozygosity (*H*_e_) and Wright's inbreeding coefficient (*F*_IS_)

Locus	*H*_e_						*F*_IS_				
	Wild	A	F1	F2o	F2s	F2c	A	F1	F2o	F2s	F2c
GER07	0.901	0.827	0.907	0.869	0.667	0.868	0.814*	0.888*	0.560*	1.000 ns	−0.045 ns
GER29	0.410	0.403	0.398	0.596	0.000	0.500	0.809*	0.815*	0.753*	–	−0.162 ns
GER45A	0.236	0.051	0.328	0.478	0.000	0.498	1.000 ns	0.912*	0.761*	–	−0.120 ns
GER45B	0.597	0.665	0.451	0.427	0.667	0.317	0.807*	0.934*	0.933*	1.000 ns	−0.101 ns
GER08	0.746	0.616	0.799	0.646	0.667	0.628	0.792*	0.886*	0.646*	1.000 ns	0.074 ns
GER17A	0.507	0.471	0.528	0.487	0.000	0.506	0.782*	0.833*	0.413 ns	–	−0.057 ns
GER17B	0.712	0.656	0.720	0.700	0.667	0.702	0.844*	0.829*	0.633*	1.000 ns	0.039 ns
GER30	0.582	0.504	0.490	0.477	0.000	0.431	0.949*	0.790*	0.461*	–	−0.025 ns
GER26	0.637	0.492	0.705	0.759	0.667	0.713	0.844*	0.902*	0.830*	1.000 ns	0.380*
GER05	0.729	0.541	0.798	0.823	0.750	0.753	0.763*	0.889*	0.722*	0.667 ns	−0.080 ns
GER42A	0.182	0.148	0.202	0.056	0.000	0.131	1.000*	0.781*	−0.015 ns	–	−0.063 ns
GER42B	0.037	0.100	0.000	0.000	0.000	0.000	1.000*	–	–	–	–
GER41	0.755	0.619	0.806	0.681	0.667	0.726	0.793*	0.892*	0.748*	1.000 ns	−0.088 ns
GER23	0.585	0.520	0.620	0.563	0.667	0.467	1.000*	0.908*	0.848*	1.000 ns	0.254 ns
GER27	0.716	0.686	0.659	0.574	0.667	0.487	0.888*	0.935*	0.751*	1.000 ns	−0.242 ns
GER47A	0.523	0.491	0.546	0.611	0.667	0.552	0.843*	0.892*	0.952*	1.000 ns	−0.263 ns
GER47B	0.587	0.534	0.620	0.638	0.000	0.651	1.000*	0.953*	0.585*	–	0.107 ns
GER38	0.735	0.537	0.774	0.678	0.000	0.659	1.000*	0.943*	0.826*	–	−0.306*
GER35A	0.106	0.101	0.109	0.000	0.000	0.000	1.000 ns	1.000*	–	–	–
GER35B	0.723	0.584	0.783	0.714	0.667	0.715	0.868*	0.909*	0.618*	1.000 ns	−0.269 ns
Mean	0.550	0.477	0.562	0.539	0.371	0.515	0.866*	0.891*	0.703*	0.966*	−0.049 ns

Note

Departure from Hardy–Weinberg expectations: ns: not significant; *p < .05 after Bonferroni correction.

### 3.3 Genetic structure at wide geographic scale

**FIGURE 2:**
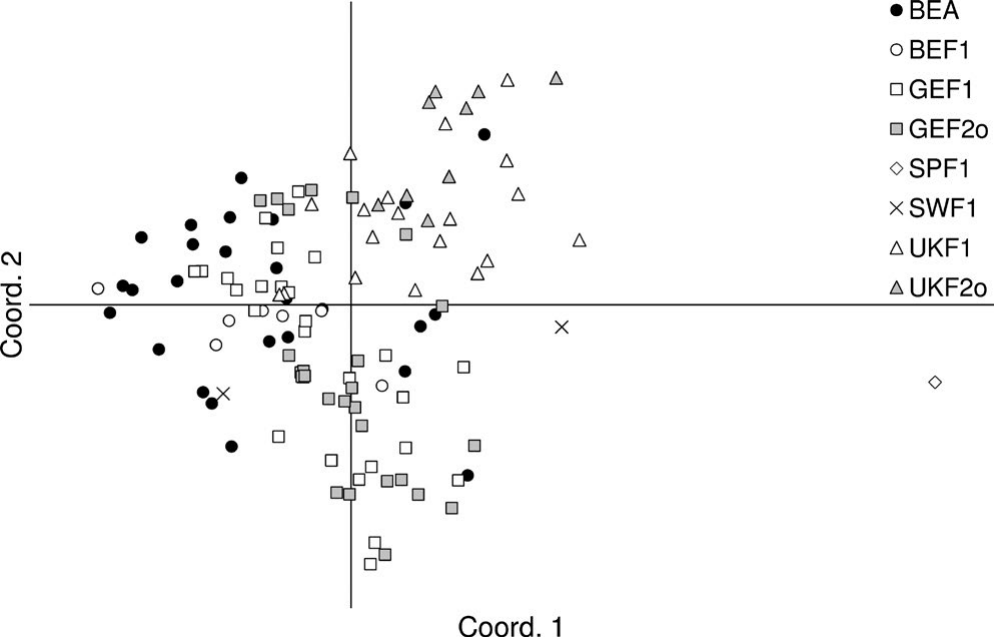
Principal coordinate analysis (PCoA) plot for 144 samples from 43 populations of *Geranium robertianum*. Axes 1 and 2 explained 13.4% and 11.7% of the total variation, respectively. Country of origin: BE: Belgium; GE: Germany, SP: Spain, SW: Sweden. Generation: A: adults; F1: F1 seed progeny; F2o: F2 seed progeny, obtained from open pollination

**FIGURE 3:**
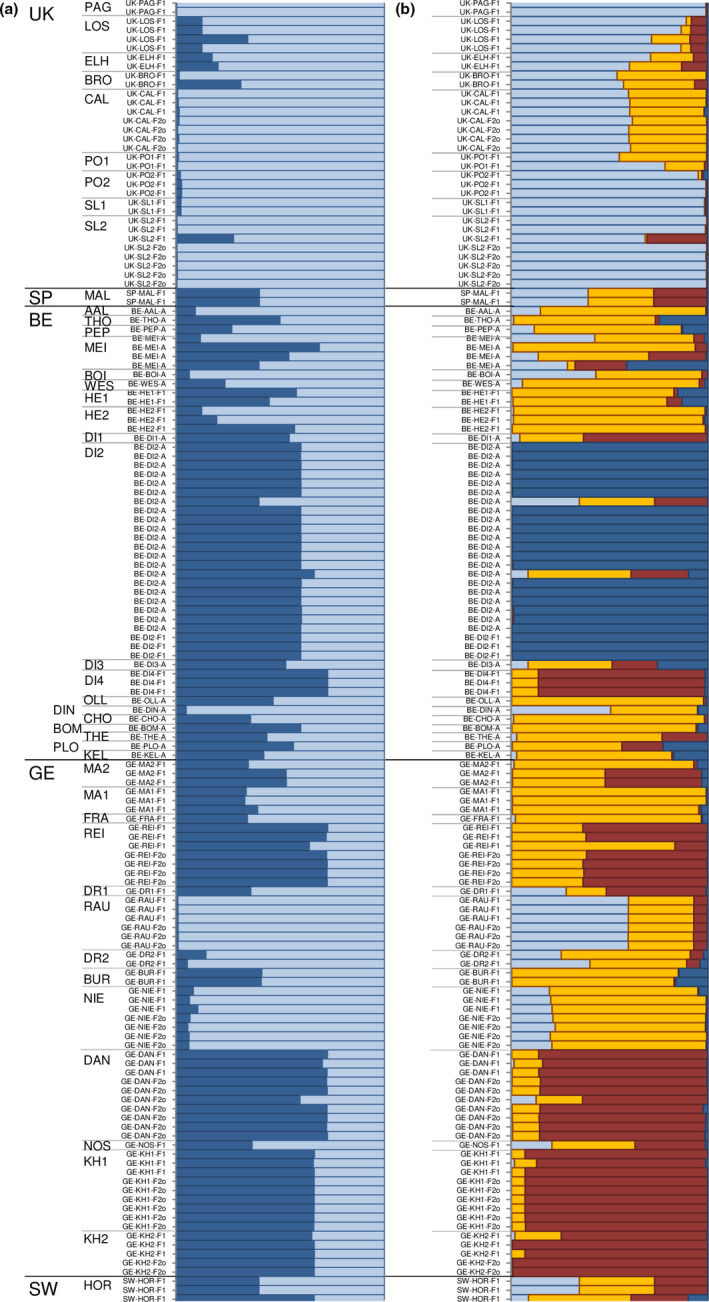
Results of Bayesian clustering (modal *K* = 2 and 4) for 144 samples from 43 populations of *Geranium robertianum* (ordered by increasing longitude). Each horizontal bar in the bar plot represents one individual and shows the probability of membership to each of the two or four clusters. Country of origin: BE: Belgium; GE: Germany, SP: Spain, SW: Sweden. Generation: A: adults; F1: F1 seed progeny; F2o: F2 seed progeny, obtained from open pollination. For population codes, see Table 1

The PCoA distinguished Spanish samples from the other populations that showed some continuous variation, although UK samples tended to be separated from Belgian, German, and Swedish samples that overlapped (Figure 2). Within each region, adult, F1, and/or F2o generations overlapped, suggesting similar mating processes in the three generations. The Bayesian clustering analysis gave an optimal number of clusters at *K* = 2. The UK samples showed high membership (*Q*) values for cluster 1 (≥80% for 91% of the individuals) and clustered together with a few German and Belgian samples (e.g., AAL, BOI, DIN, DR2, HE2, NIE, and RAU) (Figure 3a). A second peak was found for Delta*K* at *K* = 4, further distinguishing the DI2 population from Belgium (from which there were 21 samples) and some longitudinal trend for the continental populations (Figure 3b). The clustering was not related to habitat differences (Figure 1, Table 1).

## 4 DISCUSSION

The highly positive inbreeding coefficient (*F*_IS_) values found for adults, F1, and F2o progeny supported the former hypothesis (Bertin, 2001; Yeo, 1985) that autonomous self‐pollination is the main mating process contributing to seed production in wild populations of *G. robertianum*, and that outcross pollination was limited, despite the presence of attractive signals for insect pollination such as nectar production (Endress, 2010) and reporting of pollinator visitations (Bertin, 2001; Tofts, 2004). However, given the high number of flowers per plant, geitonogamous self‐pollination might also be possible in case of pollinators visiting several flowers on the same plant (Goodwillie et al., 2010; Richards, 1997). Moreover, crosses between closely related individuals, such as full siblings with the same multilocus genotype, resulting in biparental inbreeding, might also contribute to high *F*_IS_ values (Bomblies et al., 2010). This needs to be verified by investigating within‐population genetic variation with more samples (Leipold et al., 2020). Spontaneous autonomous selfing is often observed in annuals, weeds, and pioneer species such as *G. robertianum*, whereas outcrossing is more common in perennials and species occurring in stable vegetation communities (Bartoš et al., 2020; Charlesworth, 2006). For predominantly selfing species, outcrossing rates can also vary along the flowering season, depending on pollinator and resource availability (Jullien et al., 2021).

Some genetic differentiation among samples was found, but with no pronounced geographic pattern except for the UK and Spanish (Mallorca) samples and some western–eastern longitudinal trend. Long‐distance seed dispersal (Tofts, 2004) might have contributed to the low geographic structure, as found for the bird seed‐dispersed *Juniperus communis* (Jacquemart et al., 2021) and for species showing epizoochorous seed dispersal, such as *Anthyllis vulneraria* (Helsen et al., 2015) and *Dianthus carthusianorum* (Rico & Wagner, 2016), as well as accidental introduction of seeds along with anthropogenic activities and infrastructures (Wierzbicka et al., 2014). Moreover, no evidence of reproductive isolation was found between the UK and German populations assigned to separate clusters as viable seeds and healthy plants were obtained from outcrosses (F. Vandelook, *unpublished data*). Local genetic differentiation between populations may have resulted not only from genetic drift effects promoted by spontaneous selfing, but also possibly from local ecological adaptation (Bomblies et al., 2010; Hartfield et al., 2017; Wierzbicka et al., 2014). To get a comprehensive view of genetic structure patterns and of their shaping factors, we need to expand the sampling within populations and across species' distribution range.

The presence of duplicate loci suggests that the species might be of polyploid origin, which is consistent with the hypothesis that *G. robertianum* is an allotetraploid resulting from hybridization between *G. purpureum* and another unknown parental species, based on chromosome numbers, morphological similarities, cytological observations, and nectar composition (Baker & Baker, 1976; Widler‐Kiefer & Yeo, 1987; Yeo, 1973, 2004). Tetrasomic inheritance, that is, random pairing of four homologous chromosomes, leading to all possible combinations of up to four alleles per locus, can be expected for autotetraploids (Soltis et al., 2014; Stift et al., 2008). Disomic inheritance, with two separate pairs of two homologous chromosomes, is usually found in allotetraploids, but disomic inheritance can also establish in autopolyploids when whole‐genome duplication is ancient, through the action of genetic drift combined with selection (Guo et al., 2015; Le Comber et al., 2010; Soltis et al., 2014). The fact that most microsatellite markers developed in the present study behave as diploid loci with no evidence anymore of duplication supports the hypothesis of ancient polyploidization (Yeo, 1973) and evolution to fixation of disomic inheritance in the genome of *G. robertianum*. Genetic drift and selection processes might have been promoted by the short generation times for this annual–biennial species (Tofts, 2004), autonomous self‐pollination, and the wide ecological amplitude (Le Comber et al., 2010; Soltis et al., 2014) of *G. robertianum*, which has not only colonized moist woodland habitats, but also dry railway banks, grasslands, shingles, and rock outcrops, on calcareous and acidic soils (Table 1; Tofts, 2004; Wierzbicka et al., 2014). The differences in locus duplication and the relatively high genetic diversity (Table 1) across the range of *G. robertianum* despite spontaneous autonomous selfing suggest multiple events of polyploidization (Soltis et al., 2014).

Further testing of developed molecular markers on *G. purpureum* and a comprehensive study of population genetic structure of both species might contribute to shed light on speciation processes and possible relationships between population genetic structure based on molecular markers, and morphological and environmental variation across species’ distribution range.

## CONFLICT OF INTEREST

No conflict of interest.

## AUTHOR CONTRIBUTION

**Fabienne Van Rossum:** Conceptualization (supporting); Formal analysis (lead); Methodology (supporting); Visualization (equal); Writing‐original draft (lead). **Olivier Raspé:** Conceptualization (supporting); Formal analysis (supporting); Methodology (supporting); Validation (supporting); Writing‐original draft (supporting); Writing‐review & editing (equal). **Filip Vandelook:** Conceptualization (lead); Formal analysis (supporting); Methodology (lead); Validation (equal); Visualization (equal); Writing‐original draft (equal); Writing‐review & editing (equal).

## Supporting information

Fig S1

## Data Availability

Individual multilocus genotypes are available at Zenodo (https://doi.org/10.5281/zenodo.4698869).
