# Supplementary material for: Evidence of spontaneous selfing and disomic inheritance in Geranium robertianum
Source: Ecol Evol. 2021 Jun 3;11(13):8640–53. doi: 10.1002/ece3.7677 (PMC8258199; doi:10.1002/ece3.7677)
Supplement: Supplementary file 1 — Fig S1 [file ECE3-11-8640-s001.pdf]

Supplemental Figure 1. Examples of peaks for the duplicate loci GER17, 35, 42, 45 and 47 (images copied from Geneious). The two first samples correspond to parental genotypes (F1) and the next others to the offspring genotypes (F2c, obtained after hand cross-pollination).

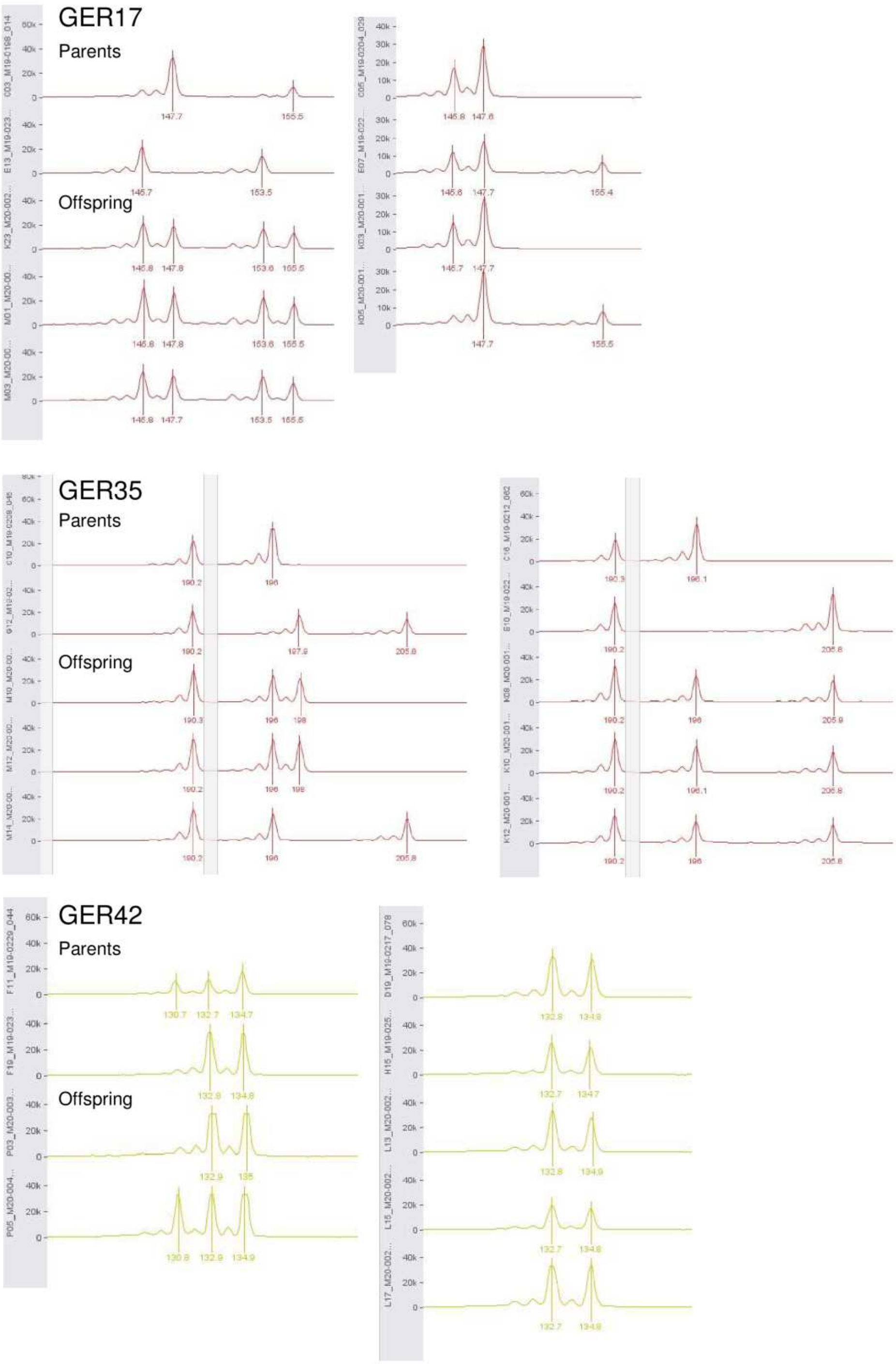

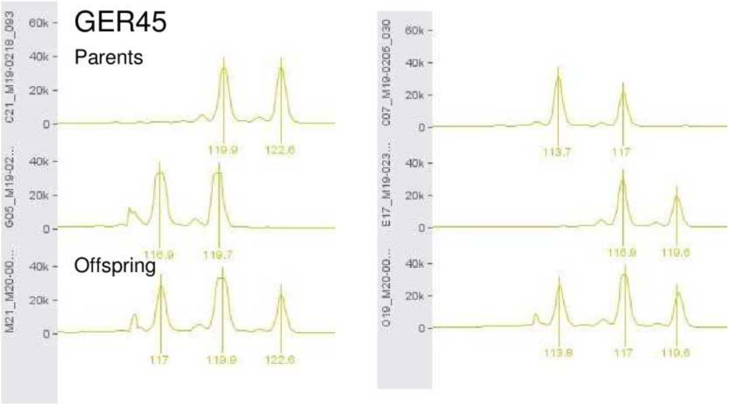
GER45
Parents
Offspring
C21_M19-0218_093
G05_M19-02...
M21_M20-00...
C07_M19-0206_030
E17_M19-023...
O19_M20-00...
119.9
122.6
116.9
119.7
117
119.9
122.6
113.7
117
116.9
119.6
113.8
117
119.6

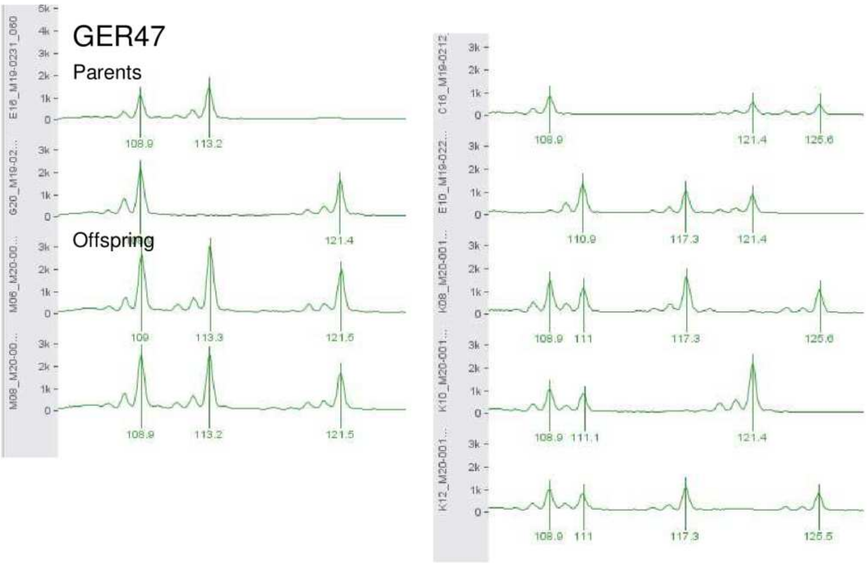
GER47
Parents
Offspring
E16_M19-0231_060
G20_M19-02...
M06_M20-00...
M08_M20-00...
C16_M19-0212
E10_M19-022...
K08_M20-001...
K10_M20-001...
K12_M20-001...
108.9
113.2
121.4
109
113.3
121.5
108.9
113.2
121.5
108.9
121.4
125.6
110.9
117.3
121.4
108.9
111
117.3
125.6
108.9
111.1
121.4
108.9
111
117.3
125.5
